# Development of Composite Semiconductor Films Based on Organotin Complexes Doped with Cobalt Porphine for Applications in Organic Diodes

**DOI:** 10.3390/ma18010045

**Published:** 2024-12-26

**Authors:** María Elena Sánchez Vergara, José Miguel Rocha Flores, Luis Alberto Cantera-Cantera, Ricardo Ballinas-Indilí, Alejandro Flores Huerta, Cecilio Álvarez-Toledano

**Affiliations:** 1 Faculty of Engineering, Universidad Anáhuac México, Av. Universidad Anáhuac 46, Col. Lomas Anáhuac, Huixquilucan 52786, Mexico; elena.sanchez@anahuac.mx (M.E.S.V.); jose_rocha@anahuac.mx (J.M.R.F.); 2 Polytechnic University of Cuautitlán Izcalli, Av. Lago de Guadalupe, Colonia Lomas de San Francisco Tepojaco, Cuautitlán Izcalli 54720, Mexico; flores2568@outlook.com; 3 Control and Automation Department, ESIME-Z, Instituto Politécnico Nacional, Unidad Profesional Adolfo López Mateos, Av. Luis Enrique Erro S/N, Gustavo A. Madero, Zacatenco, Ciudad de México 07738, Mexico; 4 Department of Chemical Sciences, Facultad de Estudios Superiores Cuautitlán Campo 1, Universidad Nacional Autónoma de México, Avenida 1o de Mayo S/N, Colonia Santa María las Torres, Cuautitlán Izcalli 54740, Mexico; ricardoballinas1989@gmail.com; 5 Chemistry Institute, Universidad Nacional Autónoma de México, Circuito Exterior S/N, Ciudad Universitaria, Ciudad de México 04510, Mexico; cecilio@unam.mx

**Keywords:** organotin complex, PEDOT:PSS, composite semiconductor film, optical properties, electrical properties

## Abstract

In this work, we present the green synthesis of complex **A**–**E** derived from β-hidroxymethylidene indanones by ultrasound, which allowed for the obtaining of compounds in a shorter time and with good yields. These organotin complexes were then doped with cobalt porphine and incorporated into a poly(3,4-ethylenedioxythiophene):poly(4-styrenesulfonate) matrix to manufacture composite semiconductor films. The semiconductor films were characterized through atomic force microscopy, examining their topography, Knoop hardness (around 17 HK), and tensile strength, which varied from 5 × 10^−4^ to 7 × 10^−2^ Pa. The optical behavior was evaluated, revealing that the changes in these characteristics are related to the type of organotin complex present in the composite film: the transmittance ranged from 77% to 86%, while the reflectance varied from 13% to 17%. The band gap, calculated using the Kubelka–Munk function F(KM), was approximately 3.7 ± 0.19 eV for all the semiconductor films. Finally, we assessed the electrical behavior of the composite films through current–voltage (I–V) measurements under different lighting conditions. The I–V curves demonstrated that they share a saturation current density of 3.46 mA/mm^2^. However, they differ in their conduction rates within the ohmic regimen. These composite films’ optical and electrical properties suggest their potential use in developing electronic devices like organic diodes.

## 1. Introduction

Poly(3,4-ethylenedioxythiophene):poly(4-styrenesulfonate), PEDOT:PSS is one of the most essential materials in organic optoelectronic devices. From a chemical point of view, PEDOT: PSS consists of positively charged conjugated conductive PEDOT and negatively charged insulating PSS [1,2,3]. The short, insoluble PEDOT chains adhere to the water-soluble long PSS chain, forming a stable grain through the Coulomb force, which allows it to remain well dispersed in water [1,2,3]. Each grain, with a 30–50 nm diameter, consists of numerous tangles. Each tangle is formed from a single PSS chain that contains several PEDOT segments. The PEDOT chains can interact with each other through orbital interactions in their central region. Additionally, hydrogen bonds formed by HSO_3_ groups between PSS layers contribute to the cohesion of the materials [3]. PEDOT:PSS stands out for its remarkable properties, including transparency, enhanced optoelectronic performance, excellent heat dissipation, and a favorable cost–benefit ratio [1]. In an optoelectronic device, the PEDOT:PSS serves as an interlayer; it provides ohmic contacts with the photoactive layer (usually with a donor polymer), photoinduced carriers (electrons or holes), and helps to tune the work function of the electrode [2]. Furthermore, when the electrical, optical, morphological, and conformational characteristics of the interlayer are controlled, the electronic performance of the optoelectronic device improves. Depending on the extraction of charge carriers, the interlayer is called the anode interlayer, or hole transport layer, which transports holes to the anode, or the cathode interlayer, which transports electrons to the cathode [2]. So, PEDOT:PSS acts as a hole transport layer (HTL), but it is not the most efficient and suitable option. One of the main problems is the stability of these devices, which limits their practical use and commercialization [4]. PEDOT’s hygroscopic and acidic nature contributes to the active layer’s degradation [3,4].

The conductivity of PEDOT:PSS is limited by the insulating character of the PSS group [3,4]. Various compounds and doping methods have been explored to enhance the efficiency of organic optoelectronic devices to modify the properties of the HTL PEDOT:PSS [4]. A notable example is graphene and its derivatives, such as graphene oxide or reduced graphene oxide [4]. These graphene derivatives increase the conductivity of PEDOT:PSS by improving hole transport and reducing the internal resistance of the device. They also create a more uniform and efficient layer for charge transport [4,5]. Graphene and its derivatives protect PEDOT against oxidation and moisture, enhancing its durability and transparency while reducing photodegradation [4,5]. Another modification of PEDOT:PSS films is the incorporation of double-walled carbon nanotubes (DWCNTs) and multi-walled carbon nanotubes (MWCNTs) [6]. Carbon nanotubes enhance electrical conductivity and form conductive networks within the polymer matrix [6]. Finally, attempts have been made to improve device efficiency by depositing biosynthesized Au-Ag nanoparticles onto PEDOT:PSS films [7]. These nanoparticles enhance the film by increasing the surface roughness of the anode. This process enlarges the interface between the active layer and the anode, creating a shorter path for holes to move toward the anode, improving hole collection [7,8]. An interesting and little-explored option is to introduce particles of organometallic semiconductors, such as organotin complexes, which will enhance the properties of PEDOT:PSS [9].

We recently reported on the conventional synthesis of organotin(IV) semiconductors derived from 2-benzylidene-1-indanone derivatives. These semiconductors have been used in the fabrication of organic optoelectronic devices. The presence of electron-donating and electron-withdrawing groups in the ligands strongly influences the electronic behavior of the devices [9]. This implies that the molecular structure and polarity of the organotin complexes can be modified by introducing peripheral substituents of diverse electronic characteristics to alter the charge transport inside the devices [10]. The possibility of enhancing the behavior of devices with PEDOT:PSS films by introducing organotin complexes, which can be modified in their chemical structure, is entirely different from the effect generated by graphene and its derivatives or carbon nanotubes or Au-Ag nanoparticles in the polymer [4,5,6,7,8]. This is due to the stable bonds formed between tin and carbon atoms, as well as with various heteroatoms. Furthermore, tin can change its coordination number, affecting the number of spaces available in its orbitals and the transport of charges within the molecule [11,12]. In organometallic semiconductors, these spaces generate preferential channels for the conduction of electrical charges through the tin atom in the molecule, and this anisotropy favors semiconductor behavior. Organotin complexes have garnered significant interest for their potential applications in optoelectronics, such as organic semiconductors, hole injectors, or active layers [9,10]. These complexes have emerged as a new family of organometallic semiconductors and have been reported to serve as sensitizers [13], PVC stabilizers [14], and active electroluminescent layers in organic light-emitting diodes (OLEDs) [15].

However, it is necessary to develop organometallic semiconductors through more efficient and environmentally friendly synthetic routes. Ultrasonic sonochemistry offers high efficiency, low energy requirements, reduced waste generation, and excellent yields. Ultrasonic cavitation can accelerate organometallic complex synthesis, providing a greener alternative to conventional methods [16]. Lately, the synthesis of the organotin amino acid Schiff bases has been efficiently carried out by ultrasonic irradiation, resulting in short reaction times and excellent yields [17]. In this work, we propose the synthesis of organotin(IV) complexes under ultrasonic irradiation, which enables the compounds to be produced in less time and with excellent yields, following environmentally friendly protocols. The organotin complexes derived can tune optical and optoelectronic properties through the chemical modulation of the ligands [18]. The optical, electrical, and electroluminescent activities of organotin complexes depend upon the number and nature of the radical groups linked to the tin ion and the anionic ligand [19,20,21,22,23,24,25]. In addition to the synthesis of organotin complexes by ultrasonic cavitation, this work introduces a novel approach to the enhancement of organometallic semiconductor films with dispersed heterojunction architecture, made of organotin complexes mechanically doped with cobalt porphine in a PEDOT:PSS matrix. An organotin–porphine pair is proposed to be embedded in a dispersed heterojunction architecture in a PEDOT:PSS matrix to test their performance in optoelectronic devices. The above is due to the adequate capacity of cobalt porphine to integrate into polymer matrices such as PEDOT:PSS [26]. To our knowledge, organotin(IV) complexes have not previously been optimized through mechanical doping; they have only been used as intrinsic semiconductors. We also proposed evaluating the performance of these films in OLED-type devices.

It is essential to consider that in the search for new families of organometallic semiconductors, some authors of this work have previously carried out studies on organometallic tin complexes; however, the differences are significant and are mainly based on the capacity of tin to present different states of coordination and on the capacity of its ligands to be incorporated into the molecular structure with different heteroatoms and types of radicals. The capacity of heptacoordinated organotin(IV) complexes as organometallic semiconductors has been studied [10]. Also, this type of coordination complex that is incorporated in PEDOT:PSS but with graphene has been studied because, without it, its semiconducting capacity is limited [27,28]. On the other hand, pentacoordinated tin complexes have been studied and embedded in PEDOT:PSS with graphene to enhance their semiconductor behavior in film [29]. Finally, complexes with 6-coordination have also been studied in this work. However, the studies have been primarily focused on the chemical structure, which has been validated by theoretical calculations [9], or on the fabrication of optoelectronic devices with PEDOT:PSS and molybdenum oxide [30]. The optoelectronic studies in all cases have varied, depending on the characteristics mentioned above, in the structure of the organotin complexes. From the above, it is clear that there is a need to continue studies in this diverse family of organometallic complexes with great potential for use in organic electronics.

## 2. Materials and Methods

The reagents were used as received: dibutyltin(IV) oxide, sodium methoxide (obtained from a commercial source (Sigma-Aldrich, Carlsbad, CA, USA)), *n*-hexane, and HPLC methanol (acquired from Merck Millipore, Steinheim, NW, Germany). The ^1^H and ^13^C NMR spectra were generated in CDCl_3_ with Bruker 300 Ascend equipment (Bruker, Ettlingen, BW, Germany) operating at 300 and 75 MHz, respectively. The 0.8% in H_2_O polymeric matrix, poly(3,4-ethylenedioxythiophene): poly(styrenesulfonate) (PEDOT:PSS: [C_8_H_8_O_3_S]-[C_6_H_6_O_2_S]_n_),2,3,7,8,12,13,17,18-octaethyl-21H,23H-porphine cobalt(II), came from a commercial source (Sigma-Aldrich, St. Louis, MO, USA).

**Scheme 1:**
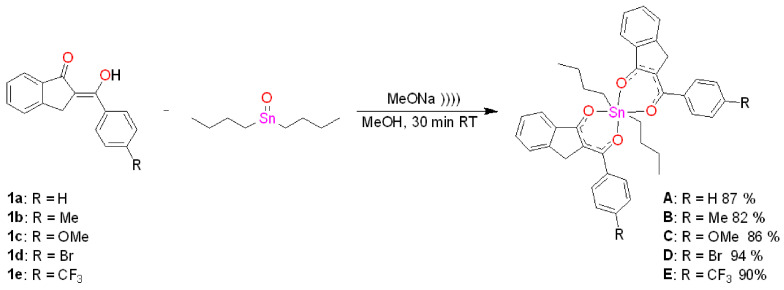
Synthesis of organotin(IV) complexes with ligands 2-benzylidene-1-indanones derivatives.

The 2-hydroxybenzylidene-1-indanone derivatives: **1a** ((*Z*)-2-(hydroxy(phenyl)methylene)-2,3-dihydro-1*H*-inden-1-one), **1b** ((*Z*)-2-(hydroxy(*p*-tolyl)methylene)-2,3-dihydro-1*H*-inden-1-one), **1c** ((*Z*)-2-(hydroxy(4-methoxyphenyl)methylene)-2,3-dihydro-1*H*-inden-1-one), **1d** ((*Z*)-2-((4-bromophenyl)(hydroxy)methylene)-2,3-dihydro-1*H*-inden-1-one), and **1e** ((*Z*)-2-(hydroxy(4-(trifluoromethyl)phenyl)methylene)-2,3-dihydro-1*H*-inden-1-one) shown in Scheme 1 were obtained according to previous reports by some of the authors of this work [9]. The dibutyltin(IV) complexes **A** (2,2-dibutyl-*bis*(4-phenyl-2,5-dihydro-1λ^3^-indeno[2,1-*e*][1,3,2]dioxa)stannine), **B** (2,2-dibutyl-bis(4-(*p*-tolyl)-2,5-dihydro-1λ^3^-indeno[2,1-*e*][1,3,2]dioxa)stannine), **C** ((2,2-dibutyl-bis(4-(4-methoxyphenyl)-2,5-dihydro-1λ^3^-indeno[2,1-*e*][1,3,2]dioxa)stannine), **D** (((2,2-dibutyl-bis(4-(4-bromorophenyl)-2,5-dihydro-1λ^3^-indeno[2,1-*e*][1,3,2]dioxa)stannine), and E (2,2-dibutyl-bis(4-(4-(trifluoromethyl)phenyl)-2,5-dihydro-1λ^3^-indeno[2,1-*e*][1,3,2]dioxa)stannine) were carried out based on a previously reported procedure with slight modification [9]. Dibutyltin(IV) oxide (0.25 g, 1.0 mmol), 2.0 equiv. of sodium methoxide and 2.0 equiv. of the corresponding 2-hydroxybenzylidene-1-indanone derivatives (**1a**–**e**) were added to a reaction tube; these reagents were dissolved in 5 mL of HPLC-grade methanol, and the reaction mixture was cavitated at room temperature for 30 min by ultrasonic irradiation at 26 KHz with the equipment at 80% of its maximum power (UP200St ultrasonic homogenizer, 200 W, 26 kHz, Hielscher Ultrasonics, Teltow, Germany). The solvent was then evaporated under reduced pressure. The respective complexes were collected by filtration and washed with minimal water and cold hexane. Complete spectroscopic characterization of organotin compounds was previously reported in the literature. Therefore, their preparation is only compared by proton and carbon NMR.

**Figure 1:**
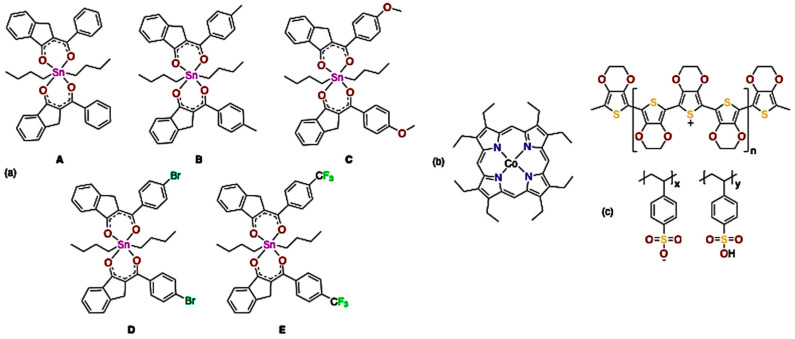
Structures of (**a**) organotin(IV) complexes mixture of isomers, (**b**) porphine cobalt (II), and (**c**) PEDOT:PSS.

The organotin(IV) semiconductors (Figure 1a) were mechanically doped with 2,3,7,8,12,13,17,18-octaethyl-21H,23H-porphine cobalt(II) (CoP: C_36_H_44_CoN_4_) (Figure 1b). Doping was carried out using a Vortex-Genie G560 shaker (Scientific Industries, Inc., Bohemia, New York, NY, USA) in a 1:1 organotin complex:cobalt porphine ratio until a homogeneous mixture was formed. This mixture was then dispersed in 0.6 mL of PEDOT:PSS (Figure 1c) using a 0.8% aqueous dispersion. The dispersion process was carried out using the vortex shaker. A Smart Coater 200 (Laurell Technologies Corporation, North Wales, PA, USA) was used to deposit the dispersed heterojunction films fabricated by the spin-coating technique. The dispersion was deposited onto the substrate, and the equipment was operated once at an angular velocity of 500 rpm for a spin time of 20 s and an acceleration of 60 rpm/s. In this experiment, 0.4 mL of dispersion was added to the substrate, and then the spin equipment was activated. Afterward, the substrate was dried on a hot plate for 2 min at 100 °C. This process was repeated two more times. Finally, the films were post-treated by exposure to isopropanol (IPA) vapor heated to 40 °C for 10 min to obtain the quinoid form of the polymer [31]. Composite films were deposited onto Corning glass slides, high resistivity monocrystalline n-type silicon wafers (c-Si), and glass covered with a transparent film of ITO (In_2_O_3_·(SnO_2_)). The Corning glass and ITO substrates were washed sequentially in an ultrasonic bath with dichloromethane, methanol, and acetone. The silicon substrates were washed with a solution of 10 mL of HF, 15 mL of HNO_3_, and 300 mL of H_2_O. The film’s topographical features, roughness, and mechanical properties were investigated in contact mode with a Nanosurf Naio atomic force microscope (Nanosurf AG, Liesta, Switzerland). UV–vis spectra were obtained with a Unicam UV300 UV-Vis 300 spectrophotometer (Thermo Fisher Scientific Inc., Waltham, MA, USA). Fluorescence was evaluated on an FP-8550 spectrofluorometer (Jasco International, Tokyo, Japan). Simple devices were fabricated for electrical characterization using ITO as the anode and silver as the cathode. A programmable voltage source, a detection station with illumination and temperature controller circuit from Next Robotix (Comercializadora KMox, S.A. de C.V., Mexico City, Mexico) and a Keithley 4200-SCS-PK1 (Tektronix Inc., Beaverton, OR, USA) automatic range sensor was used.

## 3. Results and Discussion

### 3.1. Synthesis, Deposition, and Characterization of Composite Films

The synthesis of organotin complexes was obtained by the reaction of dibutyltin(IV) oxide (1.0 equiv.) with the corresponding 2-benzylidene-1-indanone derivative (2.0 equiv.) and sodium methoxide (2.0 equiv.) in MeOH solution under ultrasonic irradiation with a short reaction time of 30 min at room temperature (see Scheme 1). The reaction had a better yield and was faster than the conventional method reported previously [9].

**Figure 2:**
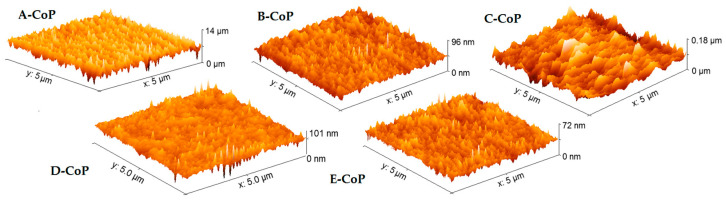
AFM images of organotin-CoP in PEDOT:PSS matrix films.

The deposited organotin-CoP in PEDOT:PSS matrix films were initially analyzed for their topography, and 3D AFM images are presented in Figure 2. As can be seen in the 3D images, all films show a relatively uniform morphology with small ridges and valleys. According to these images, the film with **C**-CoP is the most heterogeneous, and when reviewing Table 1, it can be observed that both the root mean square (RMS) and average (Ra) roughness are the highest. However, it should be noted that the roughness for all films is very low, which can guarantee, on the one hand, that spin-coating is a suitable technique for the fabrication of this type of film with dispersed heterojunction architecture. PEDOT:PSS works as an excellent matrix in this type of film since it incorporates tin complexes doped with CoP. On the other hand, the low roughness in the films will help the efficient transport of electrical charges. The homogeneity and uniformity in the films will avoid the formation of traps and with them losses in the transport of charges. Furthermore, adequate adhesion would be expected at the interface between the composite film and the electrode surface in the organic solar cell. Regarding the tensile strength (σ) shown in Table 1, variations of up to two orders of magnitude are observed. The films with organotin complexes **A**, **D**, and **E** show the highest mechanical resistance, followed by **C**, and the film with the lowest σ is the one with complex **B**. Considering that all films were manufactured under the same conditions, these results indicate that it is the type of radical in the structure of the organotin complex that is responsible for the molecular arrangement of the organotin inside the dispersed heterojunction, and with this, the value of its maximum mechanical resistance in the presence of charges. It seems that the doped **B** complex with the methyl radical is the least able to be incorporated into the PEDOT:PSS matrix, although in terms of Knoop hardness (HK), it is within the range obtained by the rest of the films. Although the film with the **D** complex presents the greatest HK, it varies very little between films, which implies that all films have similar strength, ductility and wear resistance. PEDOT:PSS functions primarily as a matrix, and its effect is to incorporate both organotin complexes and porphine and further enhance the proper morphology and topography of the films. The effect of PEDOT:PSS is directly related to the mechanical properties of organotin-CoP/PEDOT:PSS films.

**Figure 3:**
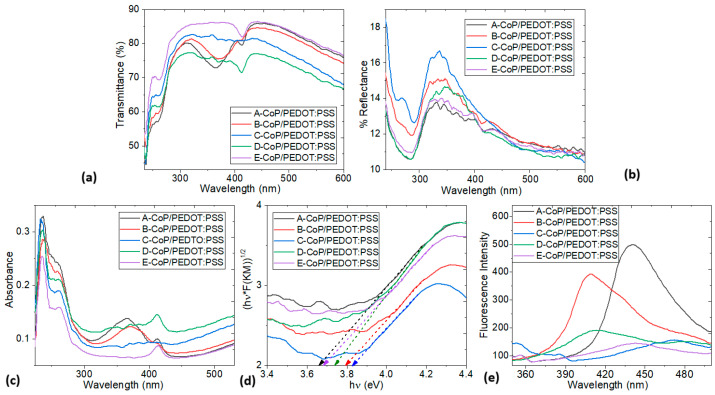
(**a**) % transmittance, (**b**) % reflectance, (**c**) absorbance, (**d**) Kubelka–Munk function, and (**e**) fluorescence of the composite films.

**Figure 4:**
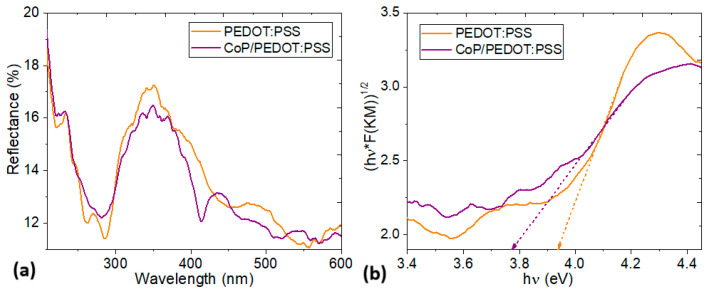
(**a**) % reflectance and (**b**) Kubelka–Munk function of the PEDOT:PSS and CoP/PEDOT:PSS films.

The spectral behavior of the transmittance, reflectance, and absorbance for the doped organotin complexes is shown in Figure 3. The transmittance, T spectrums in Figure 3a, can be split into two regions; in the first region at λ < 412 nm, an increase in T is observed until reaching a maximum, the lowest is 77.5% for the film with the **D** complex, and the highest of 86.5% is for the film with the **E** complex. Subsequently, except for the film with complex **A**, where two peaks are presented, in the rest of the films, an absorption peak is presented, after which the T increases again until reaching its maximum value and finally decreases as the λ. The absorption zones correspond to the Q-band of the CoP dopant [32]. They are interpreted as the excitation between bonding and antibonding molecular orbital in the porphine in terms of π-π* [32]. In particular, the transmittance of over 80% in most films indicates their potential to be used on surfaces that require visibility or transparency. Apparently, they are semiconductor films that can conduct electricity, like silicon-based inorganic semiconductors, but without blocking light at wavelengths that depend on the type of organotin complex that integrates the film. On the other hand, in Figure 3b, it can be observed that all the films present a Reflectance R below 16.7%, which is considered very low, and this shows the potential of the composite films to be used in optoelectronic devices. The above is because the R is an essential optical parameter in organic electronics. For instance, in OLEDs, a high R inside the device can cause unwanted reflections and reduce image contrast; however, in this case, R is very low for all films. When comparing these results with those obtained for pristine PEDOT:PSS and PEDOT:PP/CoP films (Figure 4a), it is observed that, except for the C-complex film, the rest of the organotin complex films show maximum reflectances slightly lower than those obtained for the PEDOT:PSS and CoP/PEDOT:PSS films (see Table 2). These results indicate the positive effect that organotin complexes can have as components in optoelectronic devices where low reflectance is required, as with OLEDs.

Regarding absorbance, A, in Figure 3c, an absorption band for all the spectrums is obtained around 365–415 nm, corresponding to the π-π* transition in the near-UV region of the CoP [32]. A band around 260 nm can be assigned to a π → π* intraligand transition and at 253 nm may correspond to a π → π* transition in the enolate ring [33]. The π interaction in the enolate ring is influenced by the type of substituent, as manifested in the observed spectral differences [34]. The low absorbance in the films generates positive aspects such as the lower generation of heat due to the absorption of fewer photons and the reduction in electronic noise due to the decrease in charge carriers; however, it can also decrease semiconductor behavior. For this reason, the band gap of each composite film must be evaluated, and in this work, it is proposed to use the Kubelka–Munk function F(KM) [35]. This function was obtained from the data of the R spectrum, and the value of the band gap can be estimated from the Tauc Equation (1) [35,36]:

(1) $$h\nu \alpha =A{\left(h\nu -E_{g}\right)}^{p}$$

where hν is the photon energy, α is the absorption coefficient, A is a proportionality constant, E_g_ is the forbidden energy band, and the exponent p depends on the band structure of the semiconductor. P = 2 for allowed transitions near an indirect gap where the participation of phonons is required [37]. The coefficient α is directly proportional to F(KM), and in the Tauc equation, α can be replaced by the function as follows:

(2) $$\left(h\nu \times F(KM)\right)=A{\left(h\nu -E_{g}\right)}^{p}$$

The band tails are influenced near the absorption edges, which R measurements cannot assess. Therefore, the procedure consists of plotting the (hν × F(KM))^1/2^ versus hv, fitting the linear portion of this curve by a straight line, in which the intercept provides the numerical value for E_g_ [35]. From the F(KM), in Figure 3d, (hv × F(KM))^1/2^ was plotted as a function of the incident photon energy, and the obtained band gap with its error interval values has been recorded in Table 2. All band gaps are similar, implying they have the same capacity as organic semiconductors in transporting electrical charges along the dispersed heterojunction films. When comparing these values with those obtained for the PEDOT:PSS and PEDOT:PSS/CoP films (see Figure 4b and Table 2), it can be observed that, although the values are in the same order of magnitude, a decrease in the band gap is generated by the presence of the organotin complexes **A**, **D**, and **E**. The films with complexes **B** and **C** have a band gap above the PEDOT:PSS/CoP film, which reflects the excellent optical properties of CoP, which influences the composite films with **B** and **C**. However, the effect of complexes **A**, **D**, and **E** due to the presence of their functional groups promotes a decrease in the band gap, complementing the effect of CoP. Compound **A**, which has no substituent and has a value of 3.66 eV, when compared to compound **B** or **C**, which have donor groups such as Me and OMe, respectively, it is observed how this value increases slightly to 3.79 and 3.83 eV, on the contrary when the substituent is an electron-withdrawing group such as CF_3_ in **E**, the KM value decreases slightly to 3.68 eV, which is consistent with the electronically opposite nature of these groups. The presence of the bromide group also reduces the band gap value with respect to the Me and OMe groups of **B** and **C** to 3.74 eV. Finally, the pristine PEDOT:PSS film presents the highest band gap, thus demonstrating the positive effect of organotin complexes and CoP in the composite films. PEDOT:PSS functions primarily as a matrix, and its effect is related to the mechanical properties rather than directly promoting the improvement in the optical behavior of organotin-CoP/PEDOT:PSS films.

On the other hand, fluorescence spectra in Figure 3e were obtained for all the composite films at 278 nm excitation. All maximum emissions of wavelengths are found: 440 nm for **A**-CoP, 409 nm for **B**-CoP, 472 nm for **C**-CoP, 478 nm for **D**-CoP, and 443 nm for **E**-CoP, which all correspond to blue light wavelengths. In this work, the highest emission intensity is exhibited by **A**-CoP, followed by **B**-CoP, then **D**-CoP, and finally, with similar intensities, **C**-CoP and **E**-CoP. The high fluorescence exhibited by films with **A** and **B** complexes is advantageous for their use in optoelectronic applications such as organic OLEDs. These devices can efficiently emit light when an electric current is applied or light excites them. The presence of CoP promotes the films’ optical and fluorescent properties; however, the tin complexes establish the type of behavior in the films.

### 3.2. Electrical Characterization of Devices

**Figure 5:**
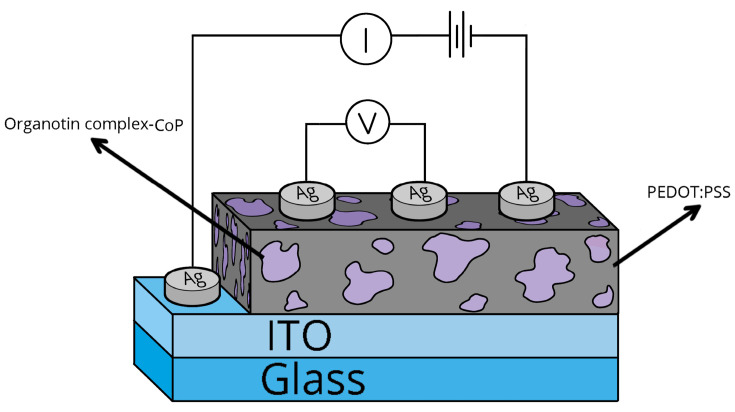
Device schematic.

**Figure 6:**
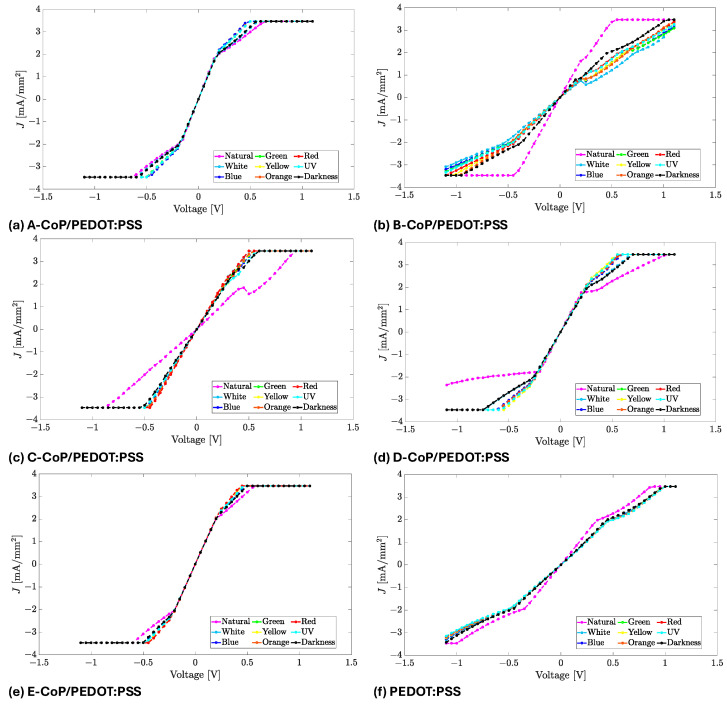
J-V curves of devices under different light conditions.

**Figure 7:**
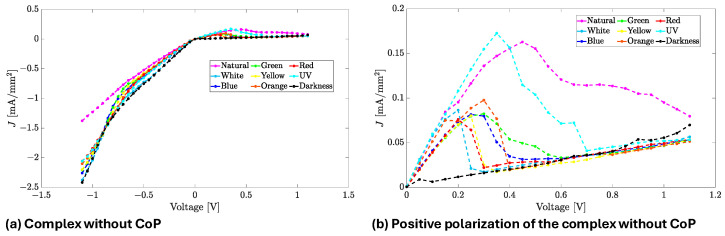
J-V curves of the complex without CoP under different light conditions.

Simple devices with the ITO/organotin complex-CoP/PEDOT:PSS/Ag conformation were fabricated to evaluate the electrical behavior of the composite films (see Figure 5). The device is rigid with a glass substrate; the ITO film acts as anode, and the four silver dots as cathode. To analyze the electrical properties of the manufactured devices, they were subjected to electrical tests under different light conditions. The tests involved applying electrical voltage ranging from −1.5 to 1.5 volts under exposure to different types of light and darkness, specifically natural, white, blue, yellow, orange, red, and ultraviolet-visible (UV) lights. Since the devices have a cross-section of 5.77 mm^2^, the devices’ current density–voltage curves (J-V) are shown in Figure 6. Additionally, Figure 7 shows the behavior of the J-V curves of the complex without CoP doping.

The behavior of the J-V curves shown in Figure 6 is generally symmetrical for the ranging voltage applied. Additionally, ohmic linear behavior is observed at low voltages. The current conduction rate changes as the voltage increases, leading to a saturation region. The saturation current density reached in all devices was 3.46 mA/mm^2^. Regarding the exposure of the devices to different types of light, the conduction rate observed changes after the linear region and in the voltage required to reach the saturation current density. It is worth noting that device **B**-CoP exhibits the most significant variations in current conduction under different types of light compared to the other devices (see Figure 6b). On the other hand, the current conduction in devices **C**-CoP and **D**-CoP under natural light does not show symmetrical behavior. Analyzing the polarization region from 0 to 1.5 volts, Table 3 shows the voltages $V_{sat}$ and $V_{ON}$ for each device and type of light exposure, where $V_{sat}$ indicates the voltage at which the devices reach the saturation current density, and $V_{ON}$ represents the voltage at which the change in the conduction rate occurs after the ohmic regime [38]. Moreover, comparing the J-V curves in Figure 6 and Figure 7 indicates that doping the complex with CoP significantly improved conductivity, achieving higher current densities.

Table 3 shows that the linear region of devices **A**-CoP to **E**-CoP lies between 0 and 0.25 V, while for the PEDOT:PSS device, it ranges from 0 to 0.4 V. Regarding the saturation voltage, the saturation current in devices **A**-CoP, **C**-CoP, **D**-CoP, and **E**-CoP is reached between 0.45 and 0.6 V, while in the PEDOT:PSS device, it is reached at 1 V. For device **B**-CoP, the saturation current is only achieved under natural light exposure and in darkness at 0.55 and 1.05 V, respectively. When other types of light were applied, saturation was not reached within the voltage range used in the experiment. On the other hand, based on the equation that describes the transition voltage between the ohmic and Space-Charge-Limited Current (SCLC) regimes in a semiconductor, $V_{ON}$, it is possible to approximate the free carrier density $n_{0}$ for each device using Equation (3).

(3) $$n_{0}=\frac{9V_{ON}\varepsilon_{0}\varepsilon_{r}}{8qd_{s}^{2}}$$

where q is the electronic charge, $d_{s}$ is the device’s thickness, $\varepsilon_{0}$ is the vacuum permittivity, and $\varepsilon_{r}$ is the dielectric constant. Applying Equation (3) with $V_{ON}=\{0.15,\;0.20,\;0.25,\;0.40\}$ from Table 3, and considering that $q=1.6\times 10^{-19}C$, $\varepsilon_{0}=8.85418\times 10^{-12}\frac{C}{\mathrm{Vm}}$, $d_{s}=8.5\times 10^{-7}m,$ and the dielectric constant is $\varepsilon_{r}=3.234$, Table 4 presents the free carrier density approximation for each device.

As shown in Table 4, the free carrier density increases as the voltage $V_{ON}$ increases; hence, the order of magnitude of the approximations in devices **A**-CoP to **E**-CoP is $10^{19}$, while the PEDOT:PSS device has a higher free carrier density on the order of $10^{20}$. One of the most notable aspects of all fabricated devices is their linear ohmic regime. In this regard, it is evident that the conduction rates in this regime differ between devices (see Figure 6). The approximation of conduction rates in the ohmic regime from 0 to $V_{ON}$ volts for each device under each type of light used and the root mean squared error (RMSE) of the approximation are shown in Table 5.

**Figure 8:**
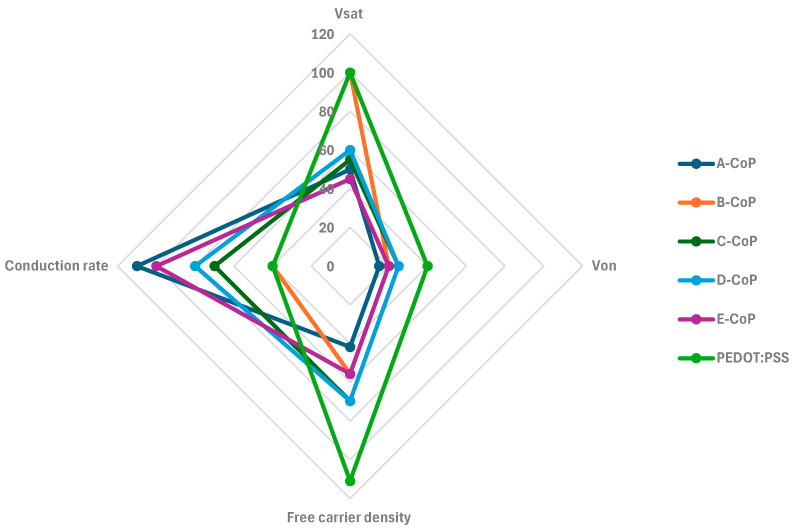
Comparison of the electrical characteristics of devices.

According to Table 5, the devices with the highest conduction rate in the ohmic regime are **A**-CoP and **E**-CoP, while devices **C**-CoP and **D**-CoP show an intermediate conduction rate. On the other hand, devices **B**-CoP and PEDOT:PSS exhibit the lowest conduction rates in this regime. Based on the results from Table 3, Table 4 and Table 5, each fabricated device exhibits different electrical characteristics, such as the conduction rate in the ohmic regime, the free carrier density $n_{0}$, the saturation voltage $V_{sat}$, and the transition voltage $V_{ON}$ between the ohmic and SCLC regimes. Figure 8 shows a comparative graph of these electrical properties.

To create the graph in Figure 8, the variables $V_{sat}$, $V_{ON}$, $n_{0}$, and conduction rate were multiplied by 100, 100, 10, and 10, respectively. Based on Figure 8, it is evident that each device has electrical properties that make them suitable for different applications. For example, the PEDOT:PSS device exhibits a wider ohmic regime than the others, indicating a high density of free carriers; however, within this ohmic region, its conduction rate is the lowest compared to devices **A**-CoP, **C**-CoP, **D**-CoP, and **E**-CoP, and it requires a voltage of 1 V to reach saturation current. On the other hand, the **B**-CoP device requires a higher voltage than the PEDOT:PSS to reach saturation current, although it has a more limited ohmic regime and a lower free carrier density. Nevertheless, both devices share the same conduction rate in the ohmic region. Devices **A**-CoP through **E**-CoP exhibit a similar ohmic regime, ranging between 0.15 and 0.25 V. However, only devices **A**-CoP, **C**-CoP, **D**-CoP, and **E**-CoP show a low saturation voltage, between 0.45 and 0.60 V. Despite having a more limited ohmic regime, these devices display a higher conduction rate than **B**-CoP and PEDOT:PSS. When comparing conduction rates in the ohmic region and saturation voltages, devices **A**-CoP and **E**-CoP stand out for having a faster transient response than the others. At the same time, PEDOT:PSS and **B**-CoP show the slowest responses. Devices **C**-CoP and **D**-CoP, on the other hand, exhibit an intermediate response.

## 4. Conclusions

Five organotin complexes were synthesized by ultrasonic irradiation, achieving excellent yields. These organotin complexes were doped with CoP and subsequently embedded in a PEDOT:PSS matrix to create compound semiconductor films. The films were characterized using atomic force microscopy (AFM) to assess their topography, roughness, hardness (HK), and tensile strength. The results indicated that the films exhibited high homogeneity with very low roughness, as evidenced by the RMS ranging from 4.5 to 17.5 nm, which enhances the films’ potential as components in devices where charge transport increases with low roughness on the surface of the films. On the other hand, the films showed a uniform hardness of around 15 HK. In contrast, the tensile strength varied significantly, ranging from 7 × 10^−2^ to 5 × 10^−4^ Pa. The type of organotin complex modified the stacking inside the films, which affected the mechanical properties. The optical properties were evaluated, revealing that changes were also related to the type of organotin complex present in the composite film: the transmittance varied from 77% to 86%, and the reflectance from 13% to 17%. The band gap obtained by the Kubelka–Munk function F(KM) was around 3.7 eV for all the films. The presence of CoP promotes the films’ optical and fluorescent properties; however, the tin complexes establish the type of behavior in the composite films. PEDOT:PSS functions primarily as a matrix, and its effect is to incorporate both organotin complexes and porphine and further enhance the proper morphology and topography of the films. The effect of PEDOT:PSS is directly related to the mechanical properties rather than directly promoting the improvement in the optical behavior of organotin-CoP/PEDOT:PSS films. The electrical tests performed on the fabricated devices showed that they all exhibit similar properties, as they share a saturation current density of 3.46 mA/mm^2^. However, they differ in their conduction rates within the ohmic regimen. Devices **A**-CoP and **E**-CoP distinguished themselves with conduction rates of 11 and 10, respectively, indicating superior conduction properties compared to the others. Regarding their electrical behavior under different light sources, most devices did not show significant changes in conduction, except for device **B**-CoP, whose conduction rate varied significantly for each type of light after the ohmic regimen. Finally, these films’ optical and electrical properties suggest they are suitable for developing electronic devices like organic diodes.

## Figures and Tables

**Table 1:** Thickness, roughness, tensile strength, and Knoop hardness of organotin-CoP in PEDOT:PSS matrix films.

Film	Thickness (µm)	RMS (nm)	Ra (nm)	σ (Pa)	HK
**A**-CoP in PEDOT:PSS matrix	6.5	11.06	8.8	8.11 × 10^−2^	14.73
**B**-CoP in PEDOT:PSS matrix	6.4	13.97	9.58	5.03 × 10^−4^	15.20
**C**-CoP in PEDOT:PSS matrix	6.2	17.48	13.47	3.77 × 10^−3^	16.55
**D**-CoP in PEDOT:PSS matrix	6.4	4.530	3.358	4.39 × 10^−2^	17.03
**E**-CoP in PEDOT:PSS matrix	6.4	4.736	3.672	7.07 × 10^−2^	13.71

**Table 2:** Maximum transmittance and Kubelka–Munk band gap of the films.

Film	Maximum Reflectance (%)	KM Band Gap (eV)
**A**-CoP in PEDOT:PSS matrix	13.8	3.66 ± 0.18
**B**-CoP in PEDOT:PSS matrix	15.2	3.79 ± 0.19
**C**-CoP in PEDOT:PSS matrix	16.7	3.83 ± 0.19
**D**-CoP in PEDOT:PSS matrix	14.6	3.74 ± 0.19
**E**-CoP in PEDOT:PSS matrix	14.0	3.68 ± 0.18
CoP in PEDOT:PSS matrix	16.5	3.77 ± 0.18
PEDOT:PSS matrix	17.3	3.94 ± 0.18

**Table 3:** Voltages for saturation and changes in the conduction rate of the devices under different light conditions.

Light	A-CoP		B-CoP		C-CoP		D-CoP		E-CoP		PEDOT:PSS	
	$V_{sat}$	$V_{ON}$	$V_{sat}$	$V_{ON}$	$V_{sat}$	$V_{ON}$	$V_{sat}$	$V_{ON}$	$V_{sat}$	$V_{ON}$	$V_{sat}$	$V_{ON}$
Natural	0.65	0.15	0.55	0.2	0.95	0.5	1.05	0.2	0.6	0.2	0.9	0.35
White	0.6	0.15	-	0.2	0.55	0.25	0.7	0.25	0.5	0.2	1	0.4
Blue	0.5	0.15	-	0.2	0.55	0.25	0.6	0.25	0.5	0.2	1	0.4
Green	0.5	0.15	-	0.2	0.55	0.25	0.6	0.25	0.45	0.2	1	0.4
Yellow	0.5	0.15	-	0.2	0.55	0.25	0.55	0.25	0.45	0.2	1	0.4
Orange	0.5	0.15	-	0.2	0.55	0.25	0.6	0.25	0.45	0.2	1	0.4
Red	0.5	0.15	-	0.25	0.5	0.25	0.6	0.25	0.45	0.2	1	0.4
UV	0.5	0.15	-	0.25	0.6	0.25	0.6	0.25	0.5	0.2	1	0.4
Darkness	0.6	0.15	1.05	0.15	0.6	0.3	0.75	0.25	0.5	0.2	1	0.4

**Table 4:** Free carrier density approximation.

Parameter.	D213	D214	D215	D216	D217	PEDOT:PSS
*V_ON_*	0.15	0.20	0.25	0.25	0.20	0.40
$n_{0}$	$4.18\times 10^{19}$	$5.57\times 10^{19}$	$6.97\times 10^{19}$	$6.97\times 10^{19}$	$5.57\times 10^{19}$	$1.11\times 10^{20}$

**Table 5:** Conduction rates in the ohmic regime for each device under different light conditions.

Light	A-CoP	RMSE	B-CoP	RMSE	C-CoP	RMSE	D-CoP	RMSE	E-CoP	RMSE	PEDOT:PSS	RMSE
Natural	11.9	$1.6\times 10^{-3}$	8.0	$6.3\times 10^{-3}$	4.4	$6.5\times 10^{-3}$	7.6	0.12	10.0	$5.0\times 10^{-3}$	5.4	0.060
White	11.2	$1.5\times 10^{-3}$	3.7	$2.9\times 10^{-3}$	7.9	$4.0\times 10^{-3}$	7.7	$1.1\times 10^{-3}$	10.0	$5.7\times 10^{-3}$	4.4	0.016
Blue	11.5	$1.6\times 10^{-3}$	4.1	$6.2\times 10^{-4}$	7.3	$5.0\times 10^{-3}$	8.0	$7.1\times 10^{-4}$	10.0	$5.0\times 10^{-3}$	4.3	0.016
Green	11.5	$1.6\times 10^{-3}$	4.2	$6.4\times 10^{-4}$	7.4	$3.8\times 10^{-3}$	8.1	$7.9\times 10^{-4}$	10.1	$5.5\times 10^{-3}$	4.3	0.016
Yellow	11.4	$1.6\times 10^{-3}$	4.2	$5.0\times 10^{-4}$	7.6	$6.0\times 10^{-3}$	8.2	$7.9\times 10^{-4}$	10.1	$4.4\times 10^{-3}$	4.3	0.016
Orange	11.4	$1.6\times 10^{-3}$	4.1	$2.0\times 10^{-3}$	7.6	$4.0\times 10^{-3}$	8.3	$7.7\times 10^{-4}$	10.1	$4.2\times 10^{-3}$	4.2	0.017
Red	11.3	$1.6\times 10^{-3}$	4.3	$1.3\times 10^{-2}$	7.9	$3.9\times 10^{-3}$	8.2	$7.0\times 10^{-4}$	10.2	$3.5\times 10^{-3}$	4.2	0.016
UV	11.3	$1.5\times 10^{-3}$	4.2	$1.0\times 10^{-2}$	6.9	$3.6\times 10^{-3}$	8.2	$7.3\times 10^{-4}$	10.0	$3.3\times 10^{-3}$	4.2	0.017
Darkness	11.2	$1.5\times 10^{-3}$	4.4	$5.8\times 10^{-2}$	7.0	$4.0\times 10^{-3}$	7.7	$1.2\times 10^{-3}$	10.0	$6.2\times 10^{-3}$	4.5	0.016

## Data Availability

Data are contained within the article.
